# Alcohol use and *APOE ε*4 interaction with cognitive domains among American adults from diverse racial/ethnic groups: A HABS-HD study

**DOI:** 10.1002/bsa3.70077

**Published:** 2026-04-14

**Authors:** Edna P. Mendoza-Villena, Malinee Neelamegam, Nicole Phillips, Leigh Johnson, Lubnaa Abdullah, James Hall, Sid O’Bryant, Eun-Young Mun, Shea J. Andrews, Robert Barber, Gita A. Pathak, Mohammad Housini, Kai Zhang, Zhengyang Zhou

**Affiliations:** 1 Department of Population and Community Health, University of North Texas Health Fort Worth, Fort Worth, Texas, USA; 2 Department of Microbiology, Immunology, and Genetics, University of North Texas Health Fort Worth, Fort Worth, Texas, USA; 3 Institute for Translational Research, University of North Texas Fort Worth, Fort Worth, Texas, USA; 4 Department of Pharmacology and Neuroscience, University of North Texas Fort Worth, Fort Worth, Texas, USA; 5 Department of Family Medicine and Osteopathic Manipulative Medicine, University of North Texas Fort Worth, Fort Worth, Texas, USA; 6 Department of Psychiatry and Behavioral Sciences, University of California San Francisco, San Francisco, California, USA; 7 The Institute for Genomic Health, Icahn School of Medicine at Mount Sinai, New York, New York, USA; 8 Department of Genetics and Genomic Sciences, Icahn School of Medicine at Mount Sinai, New York, New York, USA

**Keywords:** alcohol, apolipoprotein E *ε*4, cognitive domains, dose–response, Health and Aging Brain Study–Health Disparities, interaction

## Abstract

**Introduction:**

We investigated the interaction between alcohol use behavior and apolipoprotein E (*APOE*) *ε*4 on cognitive domains among non-Hispanic Black (NHB), Hispanic, and non-Hispanic White (NHW) individuals.

**Methods:**

Using the Health and Aging Brain Study–Health Disparities data, we analyzed 2143 adults aged 50+ using linear regression analysis with interaction between alcohol use and *APOE ε*4 on cognitive domains, stratified into NHB (*n* = 549), Hispanic (*n* = 679), and NHW (*n* = 915) groups.

**Results:**

Cognitive performance was negatively associated with alcohol use among *APOE ε*4 carriers, and this association was stronger in individuals with two *ε*4 alleles, indicating a dose-dependent effect. Such interaction was observed for executive function and episodic memory among NHB participants, and episodic memory and language among NHW participants. No significant interactions were observed among Hispanic participants.

**Discussion:**

*APOE ε*4 significantly moderated the association between alcohol use and cognitive performance of various racial/ethnic groups with a dose–response relationship.

## 1 ∣ BACKGROUND

Alcohol consumption is a modifiable risk factor for Alzheimer’s disease and related dementias (ADRD).^1^ However, the cognitive aging effects of light-to-moderate (LTM) drinking remain debated. Alcohol use can be assessed through various approaches, including quantity–frequency measures and standardized risk screening instruments. The Alcohol Use Disorders Identification Test (AUDIT), developed by the World Health Organization (WHO), is a validated 10-item instrument that assesses alcohol consumption patterns, drinking frequency, binge drinking episodes, and alcohol-related consequences.^2^ The first three items of the AUDIT (commonly referred to as AUDIT-C) specifically assess overall frequency of drinking, usual quantity of drinks consumed on drinking days, and frequency of heavy drinking.^3^ AUDIT scores of 1 to 7 indicate low-risk drinking according to WHO guidelines, while scores ≥ 8 identify hazardous or harmful alcohol use.^2^ One research study observed that individuals meeting criteria for low-risk drinking (AUDIT 1–7) predominantly self-identify as LTM drinkers,^4^ suggesting conceptual alignment between AUDIT-defined low-risk drinking and LTM drinking patterns examined in epidemiological studies. Although definitions of LTM drinking vary across studies, research examining low-risk or LTM alcohol consumption patterns has yielded inconsistent findings regarding cognitive outcomes. Zhang et al.^5^ found LTM drinking was significantly associated with a high cognitive function trajectory and a lower cognitive decline. Alternatively, Love et al.^6^ found no significant association between stable, LTM ethanol intake and cognitive decline across 15 years. Some studies indicate that LTM drinking is protective of cognitive decline,^7,8^ while others report no such association.^9-11^ Conversely, other studies report negative associations between LTM drinking and cognition.^12,13^ For example, Bos et al. reported increased risk of cognitive decline with increased alcohol intake, regardless of Alzheimer’s disease (AD) pathology.^14^

As the strongest genetic risk factor for late-onset AD, apolipoprotein E (*APOE*) *ε*4 allele carriers experience accelerated cognitive decline with age, especially in memory performance.^15,16^ Alcohol consumption is a well-recognized modifiable risk factor for cognitive impairment and decline, and genetic predisposition significantly plays a role in cognitive functions.^1^ Previous reports suggest the presence of *APOE ε*4 modifies the risk of cognitive impairment related to alcohol consumption, with *ε*4 carriers carrying an increased risk of cognitive impairment and dementia as alcohol use increases.^15,17-20^ For example, Downer et al.^19^ found that LTM alcohol consumption (1–14 drinks/week) among participants aged 65+ was linked to improvements in learning and memory among *APOE ε*4 non-carriers, but not among participants carrying one or more *APOE ε*4 alleles. Nevertheless, findings remain inconsistent. Some researchers have reported lower hazard ratios and reduced risks of poor cognitive function among *APOE ε*4 allele carriers,^21,22^ while others found no moderating effect of *APOE ε*4on alcohol use and cognition.^23-25^

The *APOE ε*4 allele is associated with a greater increase in AD risk and memory decline in individuals of European ancestry than in those with African ancestry, in whom the genetic effect is consistently weaker or absent, despite a higher frequency of the allele in Black Americans.^26^ Moreover, *APOE ε*4 positivity differs significantly across racial and ethnic groups. For example, in the Chicago Health and Aging Project, non-Hispanic Black (NHB) individuals exhibited a higher frequency of *APOE ε*4 (39%) compared to non-Hispanic White (NHW) individuals (26%).^27^ In contrast, Housini et al. found *APOE ε*4 allele frequency to be significantly higher in NHW (30%) than Hispanic (18%) individuals in the Health and Aging Brain Study–Health Disparities (HABS-HD) study.^28^ The impact of *APOE ε*4 on cognitive outcomes was found to vary across racial and ethnic groups as well.^29-31^ *APOE ε*4 carriers demonstrated lower episodic memory across all groups,^18,20^ whereas the age-related decline in semantic memory varies across NHW, Hispanic, and NHB populations.^30^

Although both alcohol use and *APOE ε*4 status are recognized as independent risk factors for impaired cognition, their interplay among NHB, Hispanic, and NHW groups has received limited attention. None of the existing studies have examined how alcohol use and *APOE ε*4 status jointly influence specific cognitive domains across these populations. Here, we assess the interaction between alcohol use and *APOE ε*4 with cognitive domains among NHB, Hispanic, and NHW participants aged 50+ to better understand how alcohol consumption and genetic risk jointly influence cognitive aging trajectories across different populations. Given the observational nature of the study sample, findings are interpreted as associations rather than causal effects. *APOE ε*4 is evaluated as a biologically motivated effect modifier of associations between alcohol use risk and cognitive domains across racial and ethnic groups. Accordingly, these analyses are exploratory within the study cohort, and results should be interpreted cautiously with respect to generalizability beyond this population.

## 2 METHODS

### 2.1 ∣ Participants

Participants for this cross-sectional study were drawn from the HABS-HD study (previously known as the Health & Aging Brain study among Latino Elders [HABLE] Study, started in 2017).^32^ The HABS-HD study, conducted at the University of North Texas Health Fort Worth (UNT Health), is an ongoing, community-based prospective cohort study that aims to examine cognitive health outcomes, including mild cognitive impairment and AD. Currently, the baseline sample size has 3343 participants. Using a community-based participatory approach, individuals aged ≥ 50 were recruited from diverse populations, including NHB, Hispanic (including Mexican American, Puerto Rican, Cuban, and other Hispanic, Latino, or Spanish origin, regardless of race), and NHW individuals, residing in the Dallas–Fort Worth (DFW) Metroplex region in Texas. The HABS-HD methods have been published elsewhere.^32^ The study operates under protocols approved by the institutional review board, with each participant or their legal representative (LAR) providing written informed consent. Researchers can access data from the HABS-HD through the Institute for Translational Research (ITR) website at UNT Health Fort Worth.^33^

In addition to the existing inclusion criteria of HABS-HD, data from this study involve participants who have available *APOE ε*4 data at baseline. To minimize bias from misreporting and reduce confounding from the sick-quitter effect, whereby individuals who stop drinking due to poor health may be misclassified as non-drinkers,^34^ the analysis was restricted to baseline participants who reported consuming alcohol at least once in the past year, as captured by the AUDIT assessment. The final analytic sample size comprised *N* = 2143 (NHB = 549 [mean age = 62.7; 38% males]; Hispanic = 679 [mean age = 62.8; 44% males]; NHW= 915 [mean age = 68.4; 44% males]).

### 2.2 ∣ Measures

#### 2.2.1 ∣ Outcomes: cognitive domains

Participants completed a battery of 11 neuropsychological tests that assessed four cognitive domains: episodic memory, executive function, processing speed, and language. Test scores representing each domain were standardized (*z* scored) and averaged to create domain scores. The performance (*z* score) is normalized using the HABS-HD cohort references of years of education (0–7, 8–12, or 13+), age (median split < 65 and > 66), primary language (English or Spanish), and ethno-racial group. Scores were reverse-coded when appropriate, such that high scores represented better performance.

Episodic memory functioning was assessed using four tests: (1) Spanish-English Verbal Learning Test (SEVLT) Learning subscale (0–60 score range, with higher scores indicating better word list learning performance),^35^ (2) SEVLT Delayed Recall subscale (0–15 score range, with higher scores indicating better delayed word list learning performance),^35^ (3) Wechsler Memory Scale, third edition (WMS-III) Logical Memory 1 subscale (0–75 score range, with higher scores indicating better immediate story recall performance),^36^ and (4) WMS-III Logical Memory 2 subscale (0–50 score range, with higher scores indicating better delayed story recall performance).^36^

Executive function was assessed using three tests: (1) WMS-III Digit Span Forward subscale (0–16 score range, with higher scores indicating better attention performance),^36^ (2) WMS-III Digit Span Backward subscale (0–14 score range, with higher scores indicating better working memory performance),^36^ and (3) Trail Making Test (TMT) Part B (scored by length of time [300 seconds] taken to complete the test, with longer times indicating worse executive functioning).^37^

Processing speed was assessed using two tests: (1) TMT Part A (scored by length of time [180 seconds] taken to complete the test, with longer times indicating worse processing speed),^37^ and (2) Digit Symbol Substitution Test (DSST; 0–93 score range, with higher scores indicating better processing speed performance).^38^

Language was assessed using two tests: (1) Animal Naming (scored by number of animal names verbalized within 60 seconds, with higher numbers indicating better semantic fluency performance),^38^ and (2) Letter Fluency (F-A-S; scored by number of words beginning with F, A, and S verbalized within 60 seconds, with higher numbers indicating better phonemic fluency performance).^39^

#### 2.2.2 ∣ Exposure: alcohol consumption

Alcohol consumption was assessed using the AUDIT, a 10-item screening instrument developed by the WHO, which was the only standardized measure of alcohol use collected in HABS-HD.^2^ The AUDIT assesses multiple dimensions of alcohol use, including consumption frequency (how often), typical quantity (how many drinks per occasion), binge drinking episodes (≥ 6 drinks on one occasion), alcohol dependence symptoms, and alcohol-related consequences or harm. Items are scored 0 to 4 (or 0, 2, and 4), with total scores ranging from 0 to 40.

According to WHO scoring guidelines, AUDIT total scores are interpreted as follows: 0 indicates abstinence; 1 to 7 indicates low-risk alcohol consumption; 8 to 14 indicates hazardous or harmful alcohol use; and ≥ 15 indicates likelihood of alcohol dependence (moderate-to-severe alcohol use disorder). The low-risk category (AUDIT 1–7) is designed to identify drinking patterns that fall within or below national low-risk drinking guidelines,^3^ which are typically set at levels below thresholds at which alcohol-related harm significantly increases.^3^

For this study, the AUDIT total score was treated as a continuous variable to capture the full range of drinking behavior within the low-risk spectrum. Only participants with AUDIT scores ≥ 1 (i.e., current drinkers reporting at least some alcohol consumption in the past year) were included in the study sample, thereby excluding lifetime abstainers and former drinkers.

#### 2.2.3 ∣ Moderator: *APOE* genotype

*APOE ε*4 carrier status was determined by genotyping the rs429358 single nucleotide polymorphism (SNP), where the C allele represents the *ε*4 risk allele. Genotypes were classified as follows: TT genotype (*ε*3/*ε*3) represents non-carriers with zero *ε*4 risk alleles; TC genotype (*ε*3/*ε*4) represents heterozygous carriers with one *ε*4 risk allele; and CC genotype (*ε*4/*ε*4) represents homozygous carriers with two *ε*4 risk alleles. For this analysis, additive coding was used to examine dose-dependent effects: 0 for TT (non-carriers), 1 for TC (heterozygous carriers), and 2 for CC (homozygous carriers).^40^ Participants lacking *APOE ε*4 data were excluded from analysis.

#### 2.2.4 ∣ Covariates

The analysis controlled for sociodemographic characteristics and depression measured by the Geriatric Depression Scale (GDS), a 30-item test used to measure self-reported depression among older adults.^41^ GDS scores were treated continuously. Sociodemographic covariates included age, sex (0 = male [reference]; 1 = female), education level (in years), and participant income (in US dollars), which was log-transformed to accommodate skewness within the data. Race/ethnicity was controlled for in the overall analysis.

### 2.3 ∣ Statistical analysis

Statistical analyses were performed using RStudio version 4.4.1. Group comparisons among NHB, Hispanic, and NHW participants were conducted using chi-squared tests for categorical variables and analysis of variance for continuous variables. Linear regression modeling with interaction terms between AUDIT and *APOE ε*4 was conducted for each cognitive domain. Analyses were performed first for the overall sample (with race/ethnicity included as a covariate), and then stratified separately for NHB, Hispanic, and NHW groups to examine potential racial/ethnic differences in the *APOE ε*4 × alcohol interaction. For each significant interaction, the relationships between the corresponding cognitive domain and AUDIT scores are plotted separately for participants with each genotype of *APOE ε*4 genotype (0, 1, and 2 risk alleles) to visualize its moderating effects. The slopes and corresponding *p* values were reported to facilitate the comparison between individuals who have 0, 1, and 2 *APOE ε*4 alleles. Regression models controlled for age, sex, income, education, first five genetic principal components (PC), and depression as covariates in all analyses, with racial/ethnic group included as an additional covariate in the overall analysis. To account for multiple comparisons across the analyses, we applied the Benjamini–Hochberg procedure to control the false discovery rate (FDR). The FDR-adjusted *p* values (*p*_FDR) were calculated separately for the overall sample and for each racial/ethnic group. Given the exploratory nature of these analyses and the a priori biological motivation for examining *APOE ε*4 × alcohol interactions across correlated cognitive domains, statistical significance was defined based on *p* values before FDR adjustment (*p <* 0.05), while FDR-adjusted results were used to assess the robustness of findings to multiple testing.

We conducted two sensitivity analyses to evaluate the robustness of our findings. First, to assess potential confounding by health and lifestyle factors, we conducted a sensitivity analysis (Sensitivity Analysis 1) that included expanded covariate adjustment beyond the primary models. Additional covariates included physical activity assessed by the Rapid Assessment of Physical Activity (RAPA) scales 1 and 2, which measure aerobic and strength/flexibility activities, respectively; body mass index (BMI, kg/m^2^); and cardiometabolic comorbidities including physician-diagnosed diabetes, hypertension, dyslipidemia, and cardiovascular disease (CVD), coded as binary variables. These covariates were selected based on their potential associations with both alcohol consumption and cognitive outcomes, as well as their availability within the HABS-HD dataset. Linear regression models with the same interaction terms as the primary analysis were fitted using this expanded set of covariates. Second, to address concerns about the skewed distribution and restricted range of AUDIT scores in our sample, we conducted a sensitivity analysis (Sensitivity Analysis 2) by categorizing alcohol consumption into low-risk drinking (AUDIT scores 1–7) and harmful/hazardous drinking (AUDIT scores ≥ 8) based on WHO guidelines. Linear regression models with the interaction terms between AUDIT categories (low-risk vs. harmful/hazardous) and *APOE ε*4 and the same covariates as the primary analyses were fitted for each cognitive domain in the overall sample and stratified by racial/ethnic group. FDR correction was applied to both sensitivity analyses as described above.

## 3 ∣ RESULTS

### 3.1 ∣ Descriptive statistics

Table 1 provides an overview of the study’s sample characteristics. The study included 2143 participants, comprising 549 NHB (25.62%), 679 Hispanic (31.68%), and 915 NHW (42.70%) individuals. Among the four cognitive domains assessed, executive function showed statistically significant differences across racial/ethnic groups (*F*[2,2140] = 6.09, *p* = 0.0023), with NHW participants showing the highest mean score (−0.02 ± 0.90) compared to NHB (−0.11 ± 0.93) and Hispanic (−0.19 ± 0.98) participants. NHW participants exhibited the highest mean episodic memory score (0.01 ± 0.87) compared to Hispanic (−0.04 ± 0.82) and NHB (−0.08 ± 0.87) participants, with the difference having no statistical significance. There were no statistically significant differences for processing speed or language.

Alcohol use, as measured by AUDIT, differed significantly by race/ethnicity (*F*[2,2140] = 11.13, *p <* 0.0001); the NHW subgroup had the highest average score (3.07 ± 2.23), followed by Hispanic (2.96 ± 2.69) and NHB (2.49 ± 2.09) subgroups. Approximately 95% of the participants have AUDIT scores ≤ 6. A significant difference in *APOE ε*4 genotype distribution was detected (*χ*^2^[3] = 89.023, *p <* 0.0001). The TT genotype was most prevalent in the Hispanic subgroup (81.15%), while the NHB subgroup had the largest proportions of TC (37.16%) and CC (5.46%) genotypes.

NHW participants were, on average, older (68.44 ± 8.75 years) than Hispanic (64.76 ± 7.44 years) and NHB (62.66 ± 7.14 years; *F*[2,2140] = 135.6, *p* < 0.0001) participants. The proportion of female participants was highest in the NHB subgroup (62.30%), followed by Hispanic (55.96%) and NHW (55.63%; *χ*^2^[2] = 7.13, *p* = 0.03) subgroups. Education attainment varied widely across groups (*F*[2,2117] = 245, *p* < 0.0001), highest among NHW (15.72 ± 2.42 years) participants, followed by NHB (14.91 ± 2.53 years) and Hispanic (12.40 ± 3.92 years) participants. Income also varied significantly (*F*[2,2042] = 46.91, *p* < 0.0001), with NHW participants reporting the highest median ($98,075), followed by NHB ($85,608) and Hispanic ($54,109) participants. Depressive symptoms, as captured by the GDS, differed significantly (*F*[2,2124] = 22.84, *p* < 0.0001), being highest in the Hispanic (6.40 ± 5.21) subgroup, followed by the NHB (5.48 ± 5.09) and NHW (4.56 ± 4.85) subgroups.

### 3.2 ∣ Regression analysis

#### 3.2.1 ∣ Alcohol use, *APOE ε*4, and cognitive performance in the overall sample

Table 2 summarizes the results of the linear regression modelling for the overall sample. Statistically significant interaction effects between AUDIT score and *APOE ε*4 were observed for episodic memory (*β* = −0.06, 95% confidence interval [CI = −0.08, −0.03], *p* < 0.001), processing speed (*β* = −0.04, 95% CI = [−0.07, −0.01], *p* = 0.004), and language (*β* = −0.03, 95% CI = [−0.06, 0.00], *p* = 0.047). However, the interaction was not statistically significant for executive function. After FDR correction for multiple comparisons, the AUDIT × *APOE ε*4 interaction remained statistically significant in the overall sample for episodic memory (*p*_FDR < 0.001), processing speed (*p*_FDR < 0.001), and language (*p*_FDR = 0.03).

**FIGURE 1:**
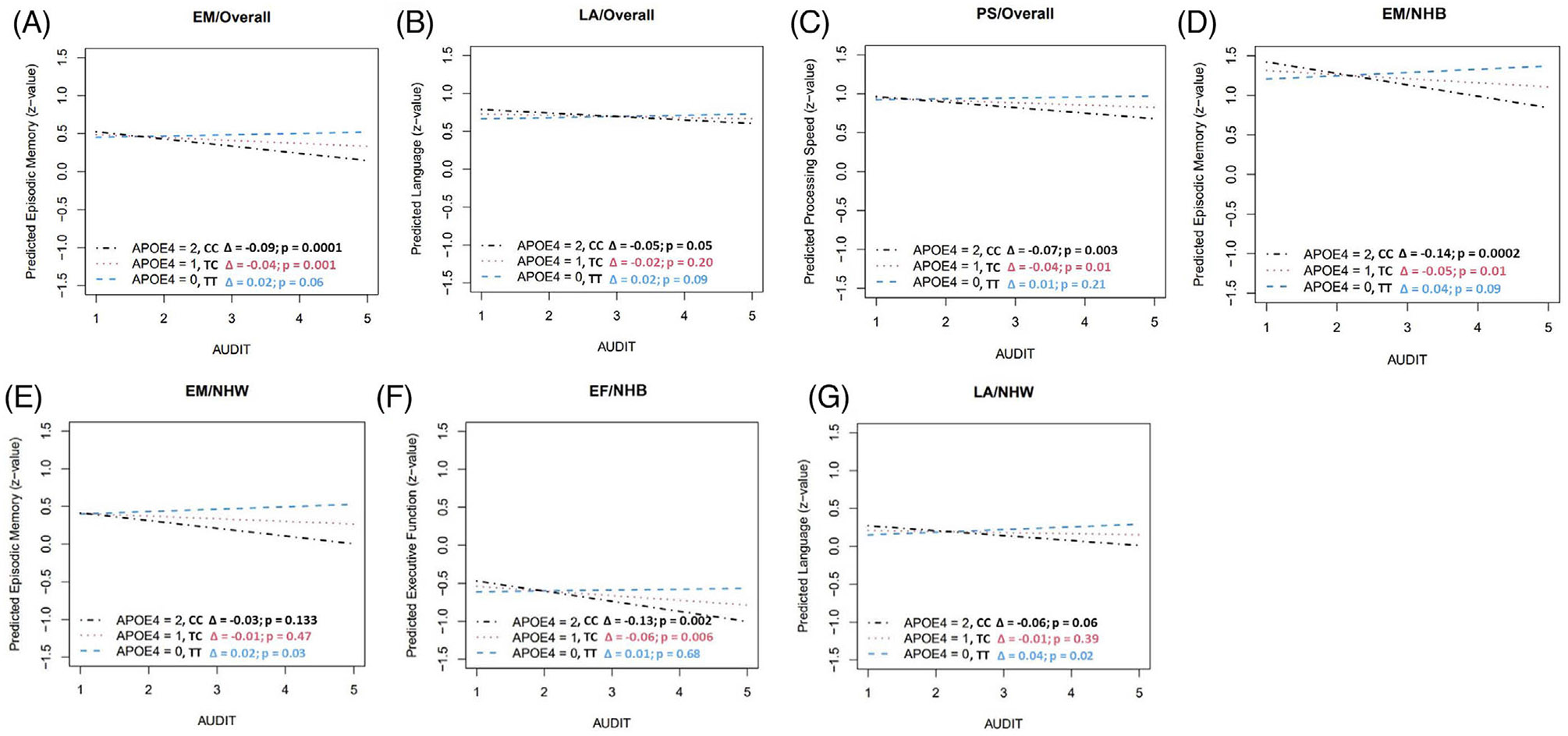
Graphical representation of the interaction between alcohol use and *APOE ε*4 allele counts on different cognitive domains. The *y* axis was set to this range for visual comparability across domains. Approximately 95% of participants have AUDIT scores between 1 and 6. A, B, C, Overall sample analysis of alcohol × *APOE ε*4 on EM, LA, and PS, respectively. D, E, Alcohol × *APOE ε*4 effect on EM among NHB and NHW. F, Alcohol × *APOE ε*4 effect on EF among NHB. G, Alcohol × *APOE ε*4 effect on LA among NHW. Black line: CC genotype (*ε*4/*ε*4) represents homozygous carriers with two *ε*4 risk alleles; red line: TC genotype (*ε*3/*ε*4) represents heterozygous carriers with one *ε*4 risk allele; and CC genotype; blue line: TT genotype (*ε*3/*ε*3) represents non-carriers with zero *ε*4 risk alleles. *APOE*, apolipoprotein E; EF, executive function; EM, episodic memory; LA, language; NHB, non-Hispanic Black; NHW, non-Hispanic White; PS, processing speed.

The relationship between alcohol use and cognitive performance for each *APOE ε*4 genotype for the overall sample can be observed in the interaction figures (Figure 1A-C). Specifically, for episodic memory (Figure 1A), *APOE ε*4 non-carriers (0 alleles) experienced a positive association with alcohol use (slope = 0.02, *p* = 0.06); however, it was not statistically significant. In contrast, this association reversed in *APOE ε*4 carriers: for individuals with one *ε*4 allele, higher AUDIT scores were associated with a significant decline in episodic memory (slope = –0.04, *p* = 0.001), and the negative association was even stronger in *ε*4 homozygotes (slope = −0.09, *p* = 0.0001), suggesting a dose-dependent association of risk.

For processing speed (Figure 1B), a similar pattern emerged: *APOE ε*4 non-carriers exhibited a non-significant positive association (slope = 0.01, *p* = 0.21), while both heterozygous (slope = −0.03, *p* = 0.01) and homozygous carriers (slope = −0.07, *p* = 0.003) experienced significant declines with increasing alcohol use, and the negative association was stronger among homozygous carriers.

In the language domain (Figure 1B), *APOE ε*4 non-carriers demonstrated a positive association (slope = 0.02, *p* = 0.09) but it was non-significant. Effects among *APOE ε*4 carriers were negative but did not reach statistical significance.

Covariate analyses revealed consistent patterns across the cognitive domains: female sex, income, and education were positively associated with cognition, while GDS scores were negatively associated with domain-specific cognitive functions.

#### 3.2.2 ∣ Alcohol use, *APOE ε*4, and cognitive performance in stratified analyses

The regression results for episodic memory of the stratified analysis are summarized in Table 3. For NHB participants, a significant interaction was observed between AUDIT score and *APOE ε*4(*β* = −0.09, 95% CI = [−0.14, −0.04], *p* < 0.001). Specifically, the relationship (Figure 1D) showed that *APOE ε*4 non-carriers demonstrated a positive association with alcohol use (slope = 0.04, *p* = 0.09), whereas *APOE ε*4 carriers showed a significant dose-dependent negative association between alcohol use and episodic memory (slope = −0.05, *p* = 0.01 for 1 allele vs. slope = −0.14, *p* = 0.0002 for 2 alleles). A similar pattern was observed for NHW participants (Figure 1F), in which *APOE ε*4 non-carriers showed a positive association between alcohol use and episodic memory (slope = 0.02, *p* = 0.03), which became negative among *APOE ε*4 carriers, especially homozygotes. No significant interaction between alcohol use and *APOE ε*4 was observed for Hispanic participants.

The regression results for executive function of the stratified analysis are summarized in Table 4. For NHB participants, the interaction between AUDIT score and *APOE ε*4 was significant (*β* = −0.07, 95% CI = [−0.13, −0.02], *p* = 0.011; Figure 1E). There was no association between alcohol use and executive function among *APOE ε*4 non-carriers, whereas both heterozygous (slope = −0.06, *p* = 0.003) and homozygous carriers (slope = −0.12, *p* = 0.003) showed a dose-dependent negative association between alcohol use and executive function. However, no statistically significant interaction effect was observed on executive function for Hispanic or NHW participants.

The regression results for processing speed of the stratified analysis are summarized in Table 5. Across the analysis for NHB, Hispanic, and NHW participants, no significant interaction between alcohol use and *APOE ε*4 was observed.

The regression results for language of the stratified analysis are summarized in Table 6. For NHW participants, the interaction between *APOE ε*4 and alcohol use was observed to be significant (*β* = −0.05, 95% CI = [−0.09, −0.01], *p* = 0.019; Figure 1G). Specifically, a positive association between alcohol use and language was observed among *APOE ε*4 non-carriers (slope = 0.04, *p* = 0.02). Negative trends between alcohol use and language were observed for *APOE ε*4 carriers, but none of the effects reached statistical significance. No significant interaction was observed for NHB and Hispanic participants.

After FDR correction, among NHB participants, the interaction remained significant for episodic memory (*p*_FDR < 0.001) and for executive function (*p*_FDR = 0.02). Among NHW participants, the interaction remained significant for episodic memory (*p*_FDR < 0.001) and for language (*p*_FDR = 0.02). No significant interactions were observed among Hispanic participants after FDR correction.

In terms of the effects of covariates across all models, female sex and higher income consistently demonstrated positive associations across cognitive domains, while higher depressive symptoms were significantly associated with poorer cognitive outcomes.

### 3.3 ∣ Sensitivity analyses

To evaluate potential confounding by physical activity, BMI, and cardiometabolic comorbidities, we conducted sensitivity analyses with expanded covariate adjustment (Sensitivity Analysis 1). The results remained largely consistent with the primary analysis. In the overall sample, significant AUDIT:*APOE ε*4 interactions were observed for episodic memory (*β* = −0.055, 95% CI = [−0.082, −0.029], *p* < 0.001, *p*_FDR < 0.001) and processing speed (*β* = −0.039, 95% CI = [−0.066, −0.012], *p* = 0.005, *p*_FDR = 0.031). Among NHB participants, the interaction remained significant for episodic memory (*β* = −0.092, 95% CI = [−0.142, −0.041], *p* < 0.001, *p*_FDR = 0.005) and marginally significant for executive function (*β* = −0.073, 95% CI = [−0.129, −0.018], *p* = 0.009, *p*_FDR = 0.056). Among NHW participants, the interaction remained significant for episodic memory (*β* = −0.069, 95% CI = [−0.109, −0.029], *p* < 0.001, *p*_FDR = 0.009) and marginally significant for language (*β* = −0.048, 95% CI = [−0.088, −0.008], *p* = 0.019, *p*_FDR = 0.074). No significant interactions were observed among Hispanic participants. Complete results are provided in Table S1 in supporting information.

In Sensitivity Analysis 2, the interaction terms between categorized AUDIT (low-risk drinking vs. harmful/hazardous drinking) and *APOE ε*4 were evaluated. In the overall sample, significant interactions were observed for episodic memory before FDR adjustment (*β* = −0.35, 95% CI = [−0.65, −0.05], *p* = 0.024, *p*_FDR = 0.246) and processing speed (*β* = −0.32, 95% CI = [−0.64, −0.01], *p* = 0.041, *p*_FDR = 0.246). Stratified analyses revealed that the interaction was most pronounced among NHB participants for episodic memory (*β* = −0.62, 95% CI = [−1.15, −0.08], *p* = 0.025, *p*_FDR = 0.152). Of note, the above results were in line with the interaction effects for episodic memory observed in our primary analyses using continuous AUDIT scores. However, none of the interaction effects were significant after FDR adjustment. No significant interactions were observed among Hispanic and NHW participants. These results are summarized in Table S2 in supporting information.

## 4 ∣ DISCUSSION

This cross-sectional study investigated the interaction between alcohol use and *APOE ε*4 status on four cognitive domains (i.e., episodic memory, executive function, processing speed, and language) among NHB, Hispanic, and NHW adults aged 50+ in the HABS-HD study. Alcohol use was assessed using the AUDIT, with scores of 1 to 7 indicating low-risk drinking. Although AUDIT reflects alcohol-related risk rather than consumption quantity, research suggests individuals in this range often self-identify as light or moderate drinkers,^3,4^ though we acknowledge these represent different measurement approaches. The vast majority of our sample (95%) had AUDIT scores ≤ 6, reflecting predominantly low-risk drinking behavior. Accordingly, our findings pertain primarily to low-risk drinking and should be interpreted with caution when extended to harmful or hazardous drinking patterns (AUDIT > 7). We conducted an overall analysis for the entire sample, and a stratified analysis for NHB, Hispanic, and NHW participants separately. The findings suggest significant interaction effects between alcohol use and *APOE ε*4 carrier status on specific cognitive domains, with notable variations across racial/ethnic groups.

In the overall sample, significant interaction effects between alcohol use and *APOE ε*4 were observed across cognitive domains. Among *APOE ε*4 carriers, low-risk alcohol use (AUDIT 1–7) was associated with poorer performance in episodic memory, processing speed, and language, whereas *APOE ε*4 non-carriers showed neutral or even positive associations. These findings align with prior studies examining LTM or low-risk drinking patterns in relation to *APOE ε*4 status. These patterns are consistent with Downer et al.,^19^ who found that low-risk/LTM alcohol consumption (1–14 drinks/week) benefitted learning and memory only in *APOE ε*4 non-carriers, and Slayday et al.,^18^ who reported similar domain-specific interactions in middle-aged adults. Our use of AUDIT-measured low-risk drinking extends these findings by demonstrating that even within the low-risk spectrum (predominantly AUDIT ≤ 6 in our sample), *APOE ε*4 carriers show negative associations with cognition while non-carriers show neutral or protective patterns. Importantly, the observed dose-dependent associations in our study, with homozygous *ε*4 carriers showing steeper declines than heterozygotes, extend these earlier observations by demonstrating a genetic gradient in alcohol-related cognitive vulnerability. Moreover, these findings remained largely robust after correction for multiple comparisons using the Benjamini–Hochberg FDR procedure, particularly for episodic memory in the overall sample and across NHB and NHW subgroups.

The mechanisms through which *APOE ε*4 may modify alcohol’s effects on cognition remain incompletely understood but could involve multiple converging pathways. *APOE ε*4 carriers appear to have greater susceptibility to cerebrovascular dysfunction, oxidative stress, and neuroinflammation compared to non-carriers.^42,43^ One possibility is that alcohol consumption may exacerbate these vulnerabilities through several pathways: by potentially impairing amyloid beta (A*β*) clearance,^44^ increasing neuroinflammatory cascades,^45^ and accelerating neurodegenerative processes.^46^ Structurally, *APOE ε*4 carriers already exhibit smaller hippocampal volumes,^47^ greater hippocampal atrophy,^48^ and increased white matter vulnerability^49^ compared to non-carriers. It is plausible that alcohol’s neurotoxic effects may further compromise these brain regions, particularly those critical for memory and executive function.^18,19^ Future studies with neuroimaging, biomarker, and longitudinal data are needed to directly examine the biological pathways underlying the observed *APOE ε*4 × alcohol interactions.

When stratified by race/ethnicity, we observed significant interactions for episodic memory and executive function among NHB participants, and episodic memory and language among NHW participants; while Hispanic participants exhibited no significant interaction effects across any cognitive domain. The absence of interactions among Hispanic participants parallels Campos et al.’s^50^ report of attenuated *APOE ε*4 effects in this population, suggesting either genetic resilience or environmental buffering. By incorporating *APOE* stratification, our study demonstrates that alcohol’s deleterious cognitive effects are neither uniform nor predictable without considering genetic risk, particularly in diverse populations in which gene–environment interactions may be moderated by ancestry-specific factors.^51^

The interaction between alcohol use and *APOE ε*4 on episodic memory was particularly robust, appearing in both the overall sample and in the NHB and NHW subgroups. This finding aligns with previous research suggesting that *APOE ε*4 carriers may be more vulnerable to memory impairments associated with alcohol consumption.^44,52^ For instance, Downer et al.^19^ reported similar findings in a study examining LTM alcohol consumption (defined as 1–14 drinks/week): low-risk drinking was associated with better learning and memory performance in *APOE ε*4 non-carriers, this benefit was absent in *APOE ε*4 carriers. Our findings, based on AUDIT-measured low-risk drinking (predominantly AUDIT ≤ 7), corroborate and extend current literature by demonstrating a dose-dependent relationship: each additional *APOE ε*4 allele amplified the negative impact of alcohol use on episodic memory. This gene dose effect suggests a biological interaction whereby alcohol may exacerbate the neural mechanisms through which *APOE ε*4 affects memory circuits, particularly in the hippocampus.^53^ Given that *APOE ε*4 carriers already demonstrate smaller hippocampal volumes and greater susceptibility to hippocampal atrophy compared to *APOE ε*4 non-carriers,^47,54^ alcohol consumption may accelerate or intensify these structural changes, especially in populations with greater genetic risk.^55,56^

The significant interaction between alcohol use and *APOE ε*4 on executive function was observed exclusively among NHB participants. This domain-specific finding contributes to the growing literature suggesting that executive function may be particularly vulnerable to combined genetic and environmental insults in certain populations.^57,58^ Executive function relies on frontal lobe integrity, and both alcohol use and *APOE ε*4 have been independently associated with alterations in prefrontal cortex circuits.^53,59,60^ Our results suggest that among NHB participants, alcohol use and *APOE ε*4 status may work together to impair executive function. *APOE ε*4 carriers are more susceptible to cerebrovascular dysfunction and oxidative stress, and alcohol use can worsen these processes by impairing A*β* clearance and increasing neuroinflammation, leading to executive function deficits.^18,61-63^ However, a previous study reported that patients of African local genomic (ALA) ancestry and are *APOE ε*4 carriers confer less risk for AD compared to *APOE ε*4 carriers of European local ancestry (ELA).^51^ This suggests that there may be other potential underlying mechanisms affecting NHB participants’ executive function such as race-based social stress or education level.^64-66^

For processing speed, significant interactions between alcohol use and *APOE ε*4 were observed in the overall sample but not in any individual racial/ethnic group. This may reflect limited statistical power in the stratified analyses or suggest that the interaction effect on processing speed is more generalizable across populations than effects on other domains. Protective associations of low-risk alcohol use in *APOE ε*4 non-carriers may result from alcohol’s potential to reduce peripheral inflammatory markers and promote metabolic homeostasis, preserving white matter microstructure.^25^ However, in *APOE ε*4 carriers, increased vulnerability to alcohol-induced white matter damage, driven by oxidative stress and neuroinflammatory cascades, leads to slower processing speed.^63^ The language domain showed significant interactions in the overall sample and among NHW participants. This pattern may stem from alcohol’s dual role in neuroinflammatory regulation combined with *APOE ε*4’s metabolic effects.^63^ In the absence of *APOE ε*4 risk alleles, low-risk alcohol use may benefit language by reducing inflammatory mediators and supporting neurotransmitter balance.^67^ Conversely, in *APOE ε*4 carriers, heightened neuroinflammation and disturbed lipid transport may impair synaptic integrity in language-related cortical regions.^63^ The absence of this interaction in NHB and Hispanic participants may indicate differential resilience or vulnerability in language networks across ethnic groups.

The robustness of our findings was evaluated using Sensitivity Analysis 1, which adjusted for additional potential confounders, including physical activity, BMI, and cardiometabolic comorbidities (diabetes, hypertension, dyslipidemia, and CVD). The interactions between AUDIT scores and *APOE ε*4 remained statistically significant or marginally significant after adjustment, with effect sizes and patterns of associations largely consistent with those observed in the primary analysis.

Results of Sensitivity Analysis 2, which used categorized AUDIT scores, were in line with the part of results for episodic memory from the primary analysis. Specifically, the interaction effect was observed to be significant (before FDR adjustment) for episodic memory among NHB participants. Of note, the limited sample sizes in the harmful/hazardous drinking category, particularly after racial/ethnic stratification (*n* = 207 overall; NHB = 55, Hispanic = 75, NHW = 77), constrained statistical power for detecting interactions in this sensitivity analysis.

The observed racial/ethnic differences in the *APOE ε*4 × alcohol interaction deserve particular attention. The most consistent interaction effects were found in NHB (episodic memory and executive function) and NHW (episodic memory and language) participants, while Hispanic participants showed no significant interactions across any domain. The above differences may partially reflect the varying prevalence of *APOE ε*4 across the racial/ethnic groups in our sample. As noted in the introduction, the NHB racial/ethnic group show a higher prevalence of *APOE ε*4 compared to NHW,^68^ while the Hispanic racial/ethnic group demonstrate a lower prevalence compared to NHW.^50^ In our sample, the proportion of *APOE ε*4 is 5.97% for Hispanic participants, which is significantly lower compared to 10.92% and 12.65% for NHB and NHW participants, respectively.

Cultural differences in drinking patterns and alcohol metabolism may influence the cognitive effects of alcohol across groups.^69^ The mean AUDIT scores of our participants differed significantly across the groups, with the NHW subgroup having the highest scores (3.07), followed by the Hispanic (2.69) and NHB subgroups (2.49). Socioeconomic factors, education differences, and health-care access may moderate the relationship between genetic risk and environmental exposures.^70,71^ In our sample, these factors varied substantially across groups, with Hispanic participants having fewer years of education and lower income levels compared to other groups. Potential genetic interactions beyond *APOE* may influence alcohol metabolism or neural resilience differently across populations.^72^ Previous research has suggested that alcohol dehydrogenase genes, which vary across ethnic groups, may moderate alcohol’s health effects.^73^

The lack of significant interactions in Hispanic participants warrants further investigation. It may reflect true biological resilience; differences in drinking patterns or alcohol metabolism; or other social, cultural, or genetic factors that mitigate the combined effects of *APOE ε*4 and alcohol on cognition in this population. Alternatively, the absence of significant interactions may be due to limited statistical power, especially if the effect sizes are smaller in this group.

Several limitations of this study should be acknowledged. The cross-sectional design precludes causal inferences about the observed associations. Longitudinal studies are needed to determine whether the observed interactions predict cognitive trajectories over time. Additionally, alcohol consumption was assessed using the AUDIT, which captures drinking patterns during the past year but may not reflect lifelong exposure or past heavy drinking episodes. Moreover, AUDIT is a self-report assessment tool and may result in underreporting of alcohol use due to social desirability bias. In addition, the vast majority of participants in this sample exhibited low-risk alcohol use (AUDIT ≤ 6), limiting the ability to draw conclusions about harmful or hazardous drinking patterns and constraining generalizability to populations with higher levels of alcohol consumption. Our sample, while diverse, was recruited from a specific geographic region of Texas (DFW Metroplex), which may limit generalizability to other regions with different demographic compositions or drinking cultures.

Despite these limitations, our study has several strengths. First, we examined the interactions between alcohol use and *APOE ε*4 across multiple cognitive domains rather than focusing on global cognition or a single domain. Second, our large, diverse, community-based sample allowed for stratified analyses across three major racial/ethnic groups of NHB, Hispanic, and NHW in the United States. Third, we used comprehensive neuropsychological assessments that captured multiple aspects of each cognitive domain.

In conclusion, our study demonstrates that *APOE ε*4 significantly moderates the relationship between alcohol use and cognitive performance across multiple domains, with carriers showing negative associations between alcohol use and cognition. These findings, based on AUDIT-measured low-risk drinking that corresponds to LTM alcohol consumption patterns examined in prior studies, suggest that the cognitive effects of low-risk alcohol use are not consistent across individuals and that genetic factors, particularly *APOE ε*4 status, significantly modify this relationship. These interaction effects vary across racial/ethnic groups, with NHB and NHW participants showing significant interactions in different cognitive domains, while no statistically significant interactions were observed among Hispanic participants.

These findings suggest that the cognitive effects of LTM alcohol consumption are not consistent across individuals, and suggest that genetic factors modify this relationship. The observed racial/ethnic differences highlight the importance of considering both genetic and sociodemographic factors when studying cognitive aging and developing interventions for diverse populations.

## Supplementary Material

S2

S1

Additional supporting information can be found online in the Supporting Information section at the end of this article.

## Figures and Tables

**TABLE 1:** Sample characteristics by race/ethnic group.

	Overall	NHB	Hispanic	NHW	Overall test
**Sample size, *n* (%)**	2143 (100)	549 (25.62)	679 (31.68)	915 (42.70)	
**Episodic memory, mean (SD)**	−0.03 (0.85)	−0.08 (0.87)	−0.04 (0.82)	0.01 (0.87)	*F*(2, 2139) = 1.95 (*p* = 0.143)
**Executive function, mean (SD)**	−0.10 (0.93)	−0.11 (0.93)	−0.19 (0.98)	−0.02 (0.90)	*F*(2, 2140) = 6.09 (*p* = 0.0023)
**Processing speed, mean (SD)**	0.04 (0.85)	0.05 (0.79)	0.02 (0.90)	0.60 (0.84)	*F*(2, 2136) = 0.499 (*p* = 0.607)
**Language, mean (SD)**	0.03 (0.82)	0.04 (0.84)	0.06 (0.82)	0.005 (0.81)	*F*(2, 2140) = 1.027 (*p* = 0.358)
**AUDIT score, mean (SD)**	2.89 (2.37)	2.49 (2.09)	2.96 (2.69)	3.07 (2.23)	*F*(2, 2140) = 11.13 (*p* < 0.0001)
***APOE*** *ε*4**_rs429358**					^2^(3) = 89.023 (*p* < 0.0001)
**Ref: TT, *n* (%)**	1510 (70.46)	315 (57.38)	551 (81.15)	644 (70.38)	
**TC, *n* (%)**	574 (26.78)	204 (37.16)	120 (17.67)	250 (27.32)	
**CC, *n* (%)**	59 (2.75)	30 (5.46)	8 (1.18)	21 (2.30)	
**Age, mean (SD)**	65.16 (8.44)	62.66 (7.14)	62.76 (7.44)	68.44 (8.75)	*F*(2, 2140) = 135.6 (*p* < 0.0001)
**Female, *n* (%)**	1231 (57.44)	342 (62.30)	380 (55.96)	509 (55.63)	^2^(2) = 7.13 (*p* = 0.03)
**Education, years (SD)**	14.46 (3.33)	14.91 (2.53)	12.40 (3.92)	15.72 (2.42)	*F*(2, 2117) = 245 (*p* < 0.0001)
**Income, median in USD**	80,939	85,608	54,109	98,075	*F*(2, 2042) = 46.91 (*p* < 0.0001)
**GDS score, mean (SD)**	5.38 (5.43)	5.48 (5.09)	6.40 (5.21)	4.56 (4.85)	*F*(2, 2124) = 22.84 (*p* < 0.0001)

*Notes*: CC genotype (*ε*4/*ε*4) represents homozygous carriers with two *ε*4 risk alleles; TC genotype (*ε*3/*ε*4) represents heterozygous carriers with one *ε*4 risk allele; and CC genotype; TT genotype (*ε*3/*ε*3) represents non-carriers with zero *ε*4 risk alleles.

Abbreviations: *APOE*, apolipoprotein E; AUDIT, Alcohol Use Disorders Identification Test; GDS, Geriatric Depression Scale; NHB, non-Hispanic Black; NHW, non-Hispanic White; SD, standard deviation; USD, US dollars.

**TABLE 2:** Regression modeling results: overall analysis across cognitive domains.

	Episodic memory (*β* estimate; 95% CI)			Executive function (*β* estimate; 95% CI)			Processing speed (*β* estimate; 95% CI)			Language (*β* estimate; 95% CI)		
	*β*	LL	UL	*β*	LL	UL	*β*	LL	UL	*β*	LL	UL
**AUDIT score**	0.02	0.00	0.04	0.00	−0.02	0.02	0.01	−0.01	0.03	0.02	0.00	0.03
***APOE*** *ε*4	0.09	−0.01	0.20	0.10	−0.01	0.22	0.06	−0.05	0.17	0.09	−0.01	0.20
**Age**	0.00	−0.01	0.00	−0.01***	−0.02	−0.01	−0.01***	−0.02	−0.01	0.00	−0.01	0.00
**Sex**	0.51***	0.43	0.58	0.09*	0.01	0.17	0.28***	0.21	0.36	0.14***	0.06	0.21
**Income**	0.02	0.00	0.05	0.06***	0.03	0.09	0.05***	0.03	0.08	0.02	0.00	0.05
**Education**	0.02*	0.00	0.03	0.02**	0.01	0.03	0.02**	0.01	0.03	0.02**	0.01	0.03
**GDS score**	−0.02***	−0.03	−0.01	−0.02***	−0.03	−0.01	−0.02***	−0.03	−0.02	−0.01***	−0.02	−0.01
**PC1**	0.15	−0.18	0.47	0.41*	0.04	0.78	0.41*	0.07	0.74	0.35*	0.01	0.68
**PC2**	0.03	−0.29	0.36	−0.13	−0.49	0.24	−0.11	−0.44	0.22	−0.13	−0.46	0.20
**PC3**	−0.15	−0.71	0.40	0.07	−0.55	0.70	0.08	−0.49	0.65	−0.16	−0.73	0.41
**PC4**	0.02	−0.31	0.34	−0.30	−0.66	0.07	−0.08	−0.42	0.25	−0.07	−0.40	0.26
**PC5**	0.02	−0.09	0.12	0.05	−0.07	0.17	0.11*	0.01	0.22	0.08	−0.02	0.19
**Race/Ethnicity: Hispanic vs. NHB**	−0.02	−0.48	0.44	−0.40	−0.91	0.11	−0.70**	−1.17	−0.23	−0.26	−0.72	0.21
**Race/Ethnicity: Hispanic vs. NHB**	−0.19	−0.66	0.27	−0.73**	−1.25	−0.21	−0.64**	−1.11	−0.17	−0.55*	−1.02	−0.08
**AUDIT:*APOE*** *ε*4	−0.06***	−0.08	−0.03	−0.03	−0.06	0.00	−0.04**	−0.07	−0.01	−0.03*	−0.06	0.00

Abbreviations: *APOE*, apolipoprotein E; AUDIT, Alcohol Use Disorders Identification Test; CI, confidence interval; GDS, Geriatric Depression Scale; LL, lower limit; NHB, non-Hispanic Black; NHW, non-Hispanic White; UL, upper limit; PC, principal component.

* *p* value < 0.05.

** *p* value < 0.01.

*** *p* value < 0.001.

**TABLE 3:** Regression modeling results: stratified analysis for episodic memory.

	NHB (*n* = 549) (*β* estimate; 95% CI)			Hispanic (*n* = 679) (*β* estimate; 95% CI)			NHW (*n* = 915) (*β* estimate; 95% CI)		
	*β*	LL	UL	*β*	LL	UL	*β*	LL	UL
**AUDIT score**	0.04	−0.01	0.09	−0.01	−0.04	0.02	0.03*	0.00	0.06
***APOE*** *ε*4	0.20*	0.02	0.38	−0.02	−0.25	0.20	0.07	−0.09	0.24
**Age**	0.00	−0.01	0.01	0.00	0.00	0.01	−0.01**	−0.02	0.00
**Sex**	0.53***	0.37	0.69	0.42***	0.29	0.55	0.56***	0.45	0.66
**Income**	−0.01	−0.06	0.04	0.02	−0.02	0.07	0.04	−0.01	0.08
**Education**	0.05***	0.02	0.08	0.00	−0.02	0.02	0.03*	0.01	0.05
**GDS score**	−0.02*	−0.03	0.00	−0.02**	−0.03	0.00	−0.03***	−0.04	−0.02
**PC1**	1.40	−0.70	3.50	0.12	−0.54	0.78	−0.66	−2.10	0.79
**PC2**	−1.89	−4.95	1.17	0.54	−0.13	1.21	−0.12	−0.54	0.30
**PC3**	−0.71	−2.95	1.53	0.12	−1.17	1.41	0.02	−0.66	0.69
**PC4**	−0.68	−2.97	1.61	−0.04	−0.76	0.67	−0.03	−0.62	0.55
**PC5**	0.15	−0.57	0.87	−0.21	−0.43	0.02	0.03	−0.10	0.16
**AUDIT:*APOE*** *ε*4	−0.09***	−0.14	−0.04	0.02	−0.04	0.08	−0.07**	−0.11	−0.03

Abbreviations: *APOE*, apolipoprotein E; AUDIT, Alcohol Use Disorders Identification Test; CI, confidence interval; GDS, Geriatric Depression Scale; LL, lower limit; NHB, non-Hispanic Black; NHW, non-Hispanic White; UL, upper limit; PC, principal component.

* *p* value < 0.05.

** *p* value < 0.01.

*** *p* value < 0.001.

**TABLE 4:** Regression modeling results: stratified analysis for executive function.

	NHB (*n* = 549) (*β* estimate; 95% CI)			Hispanic (*n* = 679) (*β* estimate; 95% CI)			NHW (*n* = 915) (*β* estimate; 95% CI)		
	*β*	LL	UL	*β*	LL	UL	*β*	LL	UL
**AUDIT score**	0.01	−0.04	0.06	−0.02	−0.06	0.01	0.02	−0.02	0.05
***APOE*** *ε*4	0.14	−0.06	0.34	0.04	−0.23	0.31	0.08	−0.10	0.26
**Age**	−0.01	−0.02	0.00	−0.01	−0.02	0.00	−0.01***	−0.02	−0.01
**Sex**	0.20*	0.02	0.37	0.00	−0.16	0.16	0.11	−0.01	0.23
**Income**	0.05*	0.00	0.11	0.10***	0.05	0.16	0.03	−0.02	0.08
**Education**	0.03	0.00	0.06	0.01	−0.01	0.03	0.03*	0.00	0.05
**GDS Score**	−0.02**	−0.04	−0.01	−0.02**	−0.03	−0.01	−0.02***	−0.04	−0.01
**PC1**	−1.33	−3.61	0.95	0.03	−0.76	0.81	−0.01	−1.60	1.58
**PC2**	2.90	−0.43	6.23	0.58	−0.21	1.38	−0.41	−0.87	0.06
**PC3**	−1.15	−3.59	1.29	1.01	−0.52	2.55	−0.01	−0.75	0.73
**PC4**	1.92	−0.58	4.41	−0.57	−1.42	0.28	−0.59	−1.24	0.06
**PC5**	0.62	−0.16	1.40	−0.14	−0.41	0.12	0.05	−0.09	0.20
**AUDIT:*APOE*** *ε*4	−0.07*	−0.13	−0.02	0.04	−0.04	0.11	−0.03	−0.07	0.02

Abbreviations: *APOE*, apolipoprotein E; AUDIT, Alcohol Use Disorders Identification Test; CI, confidence interval; GDS, Geriatric Depression Scale; LL, xxx; NHB, non-Hispanic Black; NHW, non-Hispanic White; UL, xxx.

* *p* value < 0.05.

** *p* value < 0.01.

*** *p* value < 0.001.

**TABLE 5:** Regression modeling results: stratified analysis for processing speed.

	NHB (*n* = 549) (*β* estimate; 95% CI)			Hispanic (*n* = 679) (*β* estimate; 95% CI)			NHW (*n* = 915) (*β* estimate; 95% CI)		
	*β*	LL	UL	*β*	LL	UL	*β*	LL	UL
**AUDIT score**	−0.02	−0.07	0.02	0.01	−0.02	0.04	0.02	−0.01	0.05
***APOE*** *ε*4	−0.02	−0.18	0.13	0.14	−0.11	0.39	0.04	−0.14	0.21
**Age**	−0.02***	−0.03	−0.01	−0.01	−0.02	0.00	−0.01***	−0.02	−0.01
**Sex**	0.33***	0.20	0.47	0.29***	0.14	0.43	0.25***	0.14	0.37
**Income**	0.02	−0.02	0.06	0.09***	0.04	0.14	0.04	−0.01	0.08
**Education**	0.04**	0.01	0.06	0.01	−0.01	0.03	0.02	−0.01	0.04
**GDS score**	−0.03***	−0.04	−0.02	−0.02**	−0.03	0.00	−0.03***	−0.04	−0.02
**PC1**	−0.61	−2.42	1.20	−0.07	−0.80	0.66	−0.53	−2.05	0.99
**PC2**	1.51	−1.13	4.15	0.39	−0.35	1.13	−0.43	−0.87	0.01
**PC3**	0.01	−1.93	1.94	0.69	−0.74	2.11	0.01	−0.70	0.71
**PC4**	0.62	−1.36	2.60	−0.20	−0.99	0.59	−0.25	−0.87	0.37
**PC5**	0.38	−0.24	1.00	0.03	−0.21	0.28	0.08	−0.06	0.22
**AUDIT:*APOE*** *ε*4	−0.03	−0.07	0.02	−0.05	−0.11	0.02	−0.04	−0.08	0.00

Abbreviations: *APOE*, apolipoprotein E; AUDIT, Alcohol Use Disorders Identification Test; CI, confidence interval; GDS, Geriatric Depression Scale; LL, xxx; NHB, non-Hispanic Black; NHW, non-Hispanic White; UL, xxx.

* *p* value < 0.05.

** *p* value < 0.01.

*** *p* value < 0.001.

**TABLE 6:** Regression modeling results: stratified analysis for language.

	NHB (*n* = 549) (*β* estimate; 95% CI)			Hispanic (*n* = 679) (*β* estimate; 95% CI)			NHW (*n* = 915) (*β* estimate; 95% CI)		
	*β*	LL	UL	*β*	LL	UL	*β*	LL	UL
**AUDIT score**	0.01	−0.04	0.06	0.00	−0.03	0.02	0.04*	0.01	0.06
***APOE*** *ε*4	0.06	−0.13	0.24	0.08	−0.15	0.32	0.11	−0.05	0.28
**Age**	0.00	−0.01	0.01	0.00	−0.01	0.01	0.00	−0.01	0.00
**Sex**	0.20*	0.04	0.37	0.04	−0.10	0.18	0.16**	0.06	0.27
**Income**	0.00	−0.04	0.05	0.02	−0.03	0.07	0.03	−0.02	0.07
**Education**	0.04**	0.01	0.07	0.00	−0.01	0.02	0.03*	0.01	0.05
**GDS score**	−0.01	−0.02	0.01	−0.02	−0.03	0.00	−0.02**	−0.03	−0.01
**PC1**	−0.58	−2.72	1.57	−0.02	−0.71	0.67	0.30	−1.14	1.75
**PC2**	1.12	−2.01	4.25	0.30	−0.40	1.00	−0.32	−0.74	0.10
**PC3**	−0.99	−3.27	1.30	0.30	−1.05	1.65	−0.27	−0.94	0.40
**PC4**	0.10	−2.24	2.44	−0.10	−0.85	0.65	−0.24	−0.83	0.35
**PC5**	−0.30	−1.04	0.43	0.13	−0.10	0.36	0.07	−0.06	0.20
**AUDIT:*APOE*** *ε*4	−0.04	−0.09	0.01	0.03	−0.03	0.10	−0.05*	−0.09	−0.01

Abbreviations: *APOE*, apolipoprotein E; AUDIT, Alcohol Use Disorders Identification Test; CI, confidence interval; GDS, Geriatric Depression Scale; LL, xxx; NHB, non-Hispanic Black; NHW, non-Hispanic White; UL, xxx.

* *p* value < 0.05.

** *p* value < 0.01.

*** *p* value < 0.001.

## Data Availability

The HABS-HD data used in this study can be found at: University of North Texas Health Science Center (UNTHSC) Institute for Translational Research (ITR), https://apps.unthsc.edu/itr/researchers.

## Highlights

- Apolipoprotein E (*APOE*) *ε*4 modifies the effect of alcohol use on cognition in diverse populations.
- Stronger negative impact were observed among *APOE ε*4 homozygotes (dose dependent).
- Effects varied by race/ethnicity, with executive function and episodic memory obseved among non-Hispanic Black participants and episodic memory and language non-Hispanic White participants.
- Findings underscore combined genetic and lifestyle risks for cognitive decline.

## Research-in-Context

1. **Systematic review**: The authors reviewed literature using traditional sources (e.g., PubMed). Several studies investigated associations between light-to-moderate alcohol consumption with cognitive domains, and included apolipoprotein E (*APOE*) *ε*4 as a covariate. Fewer studies investigated the interaction effect between alcohol and *APOE ε*4. However, less is known about the interaction effect between increasing *APOE ε*4 alleles and alcohol use on cognition among diverse populations.
2. **Interpretation**: Our findings demonstrated a dose–response relationship between alcohol:*APOE ε*4 and cognitive performance, and that various racial/ethnic groups are differentially impacted by this relationship. Specifically, cognitive performance was negatively associated with alcohol use among *APOE ε*4 carriers, and the association was stronger among people with two copies of alleles. Such interaction was observed for cognitive domains among non-Hispanic Blacks and non-Hispanic Whites, but not among Hispanics.
3. **Future directions**: Longitudinal study design can be conducted to obtain more robust estimates and causal inferences across different racial/ethnic groups.
