# Supplementary material for: Alcohol use and APOE ε4 interaction with cognitive domains among American adults from diverse racial/ethnic groups: A HABS-HD study
Source: Alzheimers Dement Behav Socioecon Aging. Author manuscript; Available in PMC 2026 Jun 1. (PMC13220920; doi:10.1002/bsa3.70077)
Supplement: S2 [file NIHMS2169993-supplement-S2.docx]

**Supplementary Tables 1**

**Supplementary Table 1a. Results of Sensitivity Analysis 1 for expanded covariate models: Overall analysis across cognitive domains**

|  | **Episodic Memory**  **(β estimate; 95% CI)** | | | **Executive Function**  **(β estimate; 95% CI)** | | | **Processing Speed**  **(β estimate; 95% CI)** | | | **Language**  **(β estimate; 95% CI)** | | |
| --- | --- | --- | --- | --- | --- | --- | --- | --- | --- | --- | --- | --- |
|  | **β** | **LL** | **UL** | **β** | **LL** | **UL** | **β** | **LL** | **UL** | **β** | **LL** | **UL** |
| **AUDIT Total** | 0.02* | 0.00 | 0.04 | 0.00 | -0.02 | 0.02 | 0.01 | -0.01 | 0.03 | 0.01 | 0.00 | 0.03 |
| **APOE4** | 0.10 | 0.00 | 0.21 | 0.10 | -0.02 | 0.22 | 0.05 | -0.05 | 0.16 | 0.09 | -0.02 | 0.19 |
| **Age** | 0.00 | -0.01 | 0.00 | -0.01*** | -0.02 | -0.01 | -0.01*** | -0.02 | -0.01 | 0.00 | -0.01 | 0.00 |
| **Sex (ref: Male)** | 0.52*** | 0.44 | 0.59 | 0.08 | 0.00 | 0.17 | 0.28*** | 0.20 | 0.36 | 0.12** | 0.05 | 0.20 |
| **Income** | 0.02 | 0.00 | 0.05 | 0.06*** | 0.03 | 0.09 | 0.05*** | 0.02 | 0.07 | 0.02 | -0.01 | 0.04 |
| **Education** | 0.02** | 0.00 | 0.03 | 0.02** | 0.01 | 0.04 | 0.02** | 0.00 | 0.03 | 0.02** | 0.00 | 0.03 |
| **GDS Score** | -0.02*** | -0.03 | -0.01 | -0.02*** | -0.03 | -0.01 | -0.02*** | -0.03 | -0.01 | -0.01** | -0.02 | 0.00 |
| **Physical Activity**  **(RAPA 1 Total)** | 0.00 | -0.03 | 0.02 | -0.01 | -0.04 | 0.01 | 0.01 | -0.01 | 0.04 | 0.01 | -0.01 | 0.04 |
| **Physical Activity**  **(RAPA 2 Total)** | 0.02 | -0.01 | 0.05 | 0.02 | -0.01 | 0.06 | 0.05** | 0.01 | 0.08 | 0.03** | 0.00 | 0.06 |
| **BMI** | 0.01** | 0.00 | 0.02 | 0.00 | 0.00 | 0.01 | 0.00 | 0.00 | 0.01 | 0.00 | 0.00 | 0.01 |
| **Diabetes** | 0.02 | -0.07 | 0.12 | 0.02 | -0.08 | 0.13 | -0.04 | -0.14 | 0.05 | -0.04 | -0.13 | 0.06 |
| **Hypertension** | 0.03 | -0.05 | 0.11 | -0.04 | -0.13 | 0.05 | -0.05 | -0.13 | 0.03 | -0.08* | -0.16 | 0.00 |
| **Dyslipidemia** | 0.00 | -0.08 | 0.08 | 0.00 | -0.09 | 0.08 | 0.01 | -0.07 | 0.09 | -0.02 | -0.10 | 0.06 |
| **CVD** | -0.03 | -0.17 | 0.10 | 0.03 | -0.13 | 0.18 | -0.10 | -0.24 | 0.04 | -0.03 | -0.17 | 0.11 |
| **PC1** | 0.13 | -0.20 | 0.46 | 0.41* | 0.04 | 0.79 | 0.40* | 0.06 | 0.74 | 0.35* | 0.01 | 0.69 |
| **PC2** | 0.05 | -0.27 | 0.38 | -0.13 | -0.50 | 0.24 | -0.11 | -0.45 | 0.22 | -0.14 | -0.47 | 0.19 |
| **PC3** | -0.16 | -0.72 | 0.40 | 0.09 | -0.54 | 0.72 | 0.13 | -0.44 | 0.70 | -0.17 | -0.74 | 0.40 |
| **PC4** | 0.03 | -0.30 | 0.35 | -0.30 | -0.67 | 0.07 | -0.11 | -0.44 | 0.23 | -0.07 | -0.40 | 0.26 |
| **PC5** | 0.02 | -0.08 | 0.13 | 0.06 | -0.06 | 0.17 | 0.11* | 0.00 | 0.22 | 0.07 | -0.04 | 0.18 |
| **Race/ethnicity**  **(ref: NHB)** |  |  |  |  |  |  |  |  |  |  |  |  |
| **Hispanic** | 0.02 | -0.44 | 0.48 | -0.42 | -0.94 | 0.10 | -0.73** | -1.20 | -0.26 | -0.23 | -0.70 | 0.24 |
| **NHW** | -0.15 | -0.62 | 0.32 | -0.74** | -1.27 | -0.21 | -0.69** | -1.17 | -0.21 | -0.57 | -1.05 | -0.10 |
| **AUDIT:APOE4** | -0.06*** | -0.08 | -0.03 | -0.03 | -0.06 | 0.00 | -0.04** | -0.07 | -0.01 | -0.03* | -0.06 | 0.00 |
| ***FDR p_value^a^*** | 0.00*** |  |  | 0.14 |  |  | 0.31* |  |  | 0.12* |  |  |

* p-value < 0.05 | ** p-value < 0.01 | *** p-value < 0.001

FDR p_value^a^: p-value for the interaction term after FDR adjustment

**Supplementary Table 1b. Results of Sensitivity Analysis 1 for expanded covariate models: Stratified analysis for episodic memory**

|  | **NHB (n=549)**  **(β estimate; 95% CI)** | | | **Hispanic (n=679)**  **(β estimate; 95% CI)** | | | **NHW (n=915)**  **(β estimate; 95% CI)** | | |
| --- | --- | --- | --- | --- | --- | --- | --- | --- | --- |
|  | **β** | **LL** | **UL** | **β** | **LL** | **UL** | **β** | **LL** | **UL** |
| **AUDIT Total** | 0.04 | -0.01 | 0.09 | -0.01 | -0.04 | 0.02 | 0.04* | 0.01 | 0.07 |
| **APOE4** | 0.21* | 0.02 | 0.39 | -0.01 | -0.24 | 0.22 | 0.11 | -0.06 | 0.27 |
| **Age** | 0.00 | -0.01 | 0.01 | 0.00 | 0.00 | 0.01 | -0.01* | -0.01 | 0.00 |
| **Sex (ref: Male)** | 0.52*** | 0.35 | 0.68 | 0.41** | 0.27 | 0.55 | 0.60*** | 0.48 | 0.71 |
| **Income** | -0.01 | -0.06 | 0.04 | 0.02 | -0.02 | 0.07 | 0.04 | 0.00 | 0.09 |
| **Education** | 0.05*** | 0.02 | 0.09 | 0.00 | -0.02 | 0.02 | 0.03** | 0.01 | 0.05 |
| **GDS Score** | -0.02* | -0.03 | 0.00 | -0.02** | -0.03 | -0.01 | -0.03*** | -0.04 | -0.02 |
| **Physical Activity**  **(RAPA 1 Total)** | -0.01 | -0.06 | 0.04 | 0.00 | -0.04 | 0.04 | 0.01 | -0.03 | 0.05 |
| **Physical Activity**  **(RAPA 2 Total)** | 0.02 | -0.05 | 0.08 | 0.00 | -0.05 | 0.06 | 0.03 | -0.02 | 0.07 |
| **BMI** | 0.01* | 0.00 | 0.02 | 0.01 | 0.00 | 0.02 | 0.02** | 0.01 | 0.03 |
| **Diabetes** | -0.08 | -0.26 | 0.10 | 0.09 | -0.05 | 0.23 | -0.02 | -0.20 | 0.16 |
| **Hypertension** | 0.13 | -0.06 | 0.32 | -0.03 | -0.17 | 0.10 | 0.03 | -0.08 | 0.14 |
| **Dyslipidemia** | -0.01 | -0.17 | 0.15 | 0.03 | -0.12 | 0.17 | -0.02 | -0.13 | 0.10 |
| **CVD** | -0.03 | -0.32 | 0.26 | 0.00 | -0.28 | 0.28 | -0.02 | -0.21 | 0.17 |
| **PC1** | 1.02 | -1.11 | 3.15 | 0.10 | -0.57 | 0.77 | -0.80 | -2.25 | 0.65 |
| **PC2** | -1.38 | -4.48 | 1.73 | 0.54 | -0.14 | 1.22 | -0.11 | -0.53 | 0.31 |
| **PC3** | -0.49 | -2.77 | 1.79 | 0.22 | -1.10 | 1.53 | -0.04 | -0.72 | 0.63 |
| **PC4** | -0.46 | -2.78 | 1.85 | -0.10 | -0.83 | 0.63 | -0.02 | -0.61 | 0.57 |
| **PC5** | 0.23 | -0.51 | 0.96 | -0.19 | -0.42 | 0.04 | 0.02 | -0.11 | 0.15 |
| **AUDIT:APOE4** | -0.09*** | -0.14 | -0.04 | 0.01 | -0.05 | 0.08 | -0.07*** | -0.11 | -0.03 |
| ***FDR p_value^a^*** | 0.005** |  |  | 0.83 |  |  | 0.008** |  |  |

* p-value < 0.05 | ** p-value < 0.01 | *** p-value < 0.001

FDR p_value^a^: p-value for the interaction term after FDR adjustment

**Supplementary Table 1c. Results of Sensitivity Analysis 1 for expanded covariate models: Stratified analysis for executive function**

|  | **NHB (n=549)**  **(β estimate; 95% CI)** | | | **Hispanic (n=679)**  **(β estimate; 95% CI)** | | | **NHW (n=915)**  **(β estimate; 95% CI)** | | |
| --- | --- | --- | --- | --- | --- | --- | --- | --- | --- |
|  | **β** | **LL** | **UL** | **β** | **LL** | **UL** | **β** | **LL** | **UL** |
| **AUDIT Total** | 0.01 | -0.04 | 0.07 | -0.03 | -0.06 | 0.01 | 0.02 | -0.02 | 0.05 |
| **APOE4** | 0.15 | -0.06 | 0.35 | 0.06 | -0.22 | 0.33 | 0.07 | -0.12 | 0.25 |
| **Age** | -0.01 | -0.02 | 0.00 | -0.01 | -0.02 | 0.00 | -0.01*** | -0.02 | -0.01 |
| **Sex (ref: Male)** | 0.21* | 0.03 | 0.39 | -0.02 | -0.18 | 0.15 | 0.09 | -0.04 | 0.22 |
| **Income** | 0.06* | 0.00 | 0.11 | 0.10*** | 0.04 | 0.16 | 0.03 | -0.02 | 0.08 |
| **Education** | 0.03 | 0.00 | 0.06 | 0.01 | -0.01 | 0.03 | 0.03* | 0.00 | 0.05 |
| **GDS Score** | -0.02* | -0.04 | 0.00 | -0.02** | -0.03 | -0.01 | -0.02*** | -0.04 | -0.01 |
| **Physical Activity**  **(RAPA 1 Total)** | 0.01 | -0.05 | 0.06 | -0.03 | -0.08 | 0.03 | -0.01 | -0.06 | 0.03 |
| **Physical Activity**  **(RAPA 2 Total)** | 0.02 | -0.06 | 0.09 | 0.03 | -0.03 | 0.10 | 0.01 | -0.04 | 0.06 |
| **BMI** | 0.00 | -0.01 | 0.01 | 0.01 | -0.01 | 0.02 | 0.00 | -0.01 | 0.01 |
| **Diabetes** | -0.05 | -0.25 | 0.15 | 0.08 | -0.09 | 0.25 | 0.00 | -0.20 | 0.20 |
| **Hypertension** | 0.08 | -0.13 | 0.29 | -0.02 | -0.18 | 0.14 | -0.09 | -0.22 | 0.03 |
| **Dyslipidemia** | -0.11 | -0.29 | 0.06 | 0.04 | -0.13 | 0.21 | 0.02 | -0.11 | 0.15 |
| **CVD** | 0.18 | -0.14 | 0.49 | 0.21 | -0.12 | 0.54 | -0.12 | -0.32 | 0.09 |
| **PC1** | -1.69 | -4.01 | 0.64 | 0.06 | -0.74 | 0.85 | 0.04 | -1.57 | 1.65 |
| **PC2** | 3.44* | 0.05 | 6.83 | 0.52 | -0.29 | 1.32 | -0.40 | -0.87 | 0.06 |
| **PC3** | -1.14 | -3.63 | 1.35 | 1.03 | -0.53 | 2.59 | 0.02 | -0.73 | 0.77 |
| **PC4** | 2.29 | -0.24 | 4.81 | -0.60 | -1.46 | 0.26 | -0.63 | -1.29 | 0.02 |
| **PC5** | 0.67 | -0.13 | 1.47 | -0.13 | -0.40 | 0.15 | 0.06 | -0.09 | 0.21 |
| **AUDIT:APOE4** | -0.07** | -0.13 | -0.02 | 0.03 | -0.04 | 0.11 | -0.02 | -0.07 | 0.02 |
| ***FDR p_value^a^*** | 0.06 |  |  | 0.83 |  |  | 0.37 |  |  |

* p-value < 0.05 | ** p-value < 0.01 | *** p-value < 0.001

FDR p_value^a^: p-value for the interaction term after FDR adjustment

**Supplementary Table 1d. Results of Sensitivity Analysis 1 for expanded covariate models: Stratified analysis for processing speed**

|  | **NHB (n=549)**  **(β estimate; 95% CI)** | | | **Hispanic (n=679)**  **(β estimate; 95% CI)** | | | **NHW (n=915)**  **(β estimate; 95% CI)** | | |
| --- | --- | --- | --- | --- | --- | --- | --- | --- | --- |
|  | **β** | **LL** | **UL** | **β** | **LL** | **UL** | **β** | **LL** | **UL** |
| **AUDIT Total** | -0.02 | -0.06 | 0.02 | 0.01 | -0.02 | 0.04 | 0.02 | -0.01 | 0.05 |
| **APOE4** | -0.02 | -0.18 | 0.13 | 0.15 | -0.10 | 0.40 | 0.02 | -0.16 | 0.19 |
| **Age** | -0.02*** | -0.03 | -0.01 | -0.01 | -0.02 | 0.00 | -0.01*** | -0.02 | -0.01 |
| **Sex (ref: Male)** | 0.36*** | 0.22 | 0.50 | 0.26*** | 0.11 | 0.42 | 0.23*** | 0.11 | 0.35 |
| **Income** | 0.02 | -0.02 | 0.06 | 0.08** | 0.03 | 0.14 | 0.03 | -0.02 | 0.07 |
| **Education** | 0.04** | 0.01 | 0.06 | 0.01 | -0.01 | 0.03 | 0.01 | -0.01 | 0.03 |
| **GDS Score** | -0.03*** | -0.04 | -0.01 | -0.01* | -0.03 | 0.00 | -0.03*** | -0.04 | -0.01 |
| **Physical Activity**  **(RAPA 1 Total)** | 0.05* | 0.01 | 0.09 | -0.02 | -0.07 | 0.02 | 0.01 | -0.03 | 0.05 |
| **Physical Activity**  **(RAPA 2 Total)** | 0.02 | -0.04 | 0.08 | 0.05 | -0.01 | 0.11 | 0.05 | 0.00 | 0.10 |
| **BMI** | 0.01 | 0.00 | 0.02 | 0.00 | -0.01 | 0.01 | 0.00 | -0.02 | 0.01 |
| **Diabetes** | -0.07 | -0.22 | 0.09 | -0.05 | -0.21 | 0.11 | -0.02 | -0.21 | 0.17 |
| **Hypertension** | 0.07 | -0.10 | 0.23 | -0.07 | -0.22 | 0.08 | -0.06 | -0.18 | 0.05 |
| **Dyslipidemia** | -0.04 | -0.17 | 0.10 | 0.00 | -0.16 | 0.16 | 0.03 | -0.09 | 0.15 |
| **CVD** | 0.08 | -0.17 | 0.32 | -0.02 | -0.33 | 0.29 | -0.23* | -0.42 | -0.03 |
| **PC1** | -0.83 | -2.65 | 0.98 | -0.03 | -0.77 | 0.71 | -0.38 | -1.90 | 1.15 |
| **PC2** | 1.84 | -0.81 | 4.48 | 0.32 | -0.43 | 1.07 | -0.43 | -0.87 | 0.01 |
| **PC3** | 0.03 | -1.91 | 1.98 | 0.67 | -0.79 | 2.12 | 0.10 | -0.61 | 0.81 |
| **PC4** | 0.78 | -1.19 | 2.75 | -0.21 | -1.01 | 0.59 | -0.28 | -0.90 | 0.34 |
| **PC5** | 0.36 | -0.27 | 0.99 | 0.05 | -0.20 | 0.31 | 0.08 | -0.06 | 0.22 |
| **AUDIT:APOE4** | -0.03 | -0.07 | 0.02 | -0.05 | -0.12 | 0.02 | -0.03 | -0.08 | 0.01 |
| ***FDR p_value^a^*** | 0.46 |  |  | 0.80 |  |  | 0.27 |  |  |

* p-value < 0.05 | ** p-value < 0.01 | *** p-value < 0.001

FDR p_value^a^: p-value for the interaction term after FDR adjustment

**Supplementary Table 1e. Results of Sensitivity Analysis 1 for expanded covariate models: Stratified analysis for language**

|  | **NHB (n=549)**  **(β estimate; 95% CI)** | | | **Hispanic (n=679)**  **(β estimate; 95% CI)** | | | **NHW (n=915)**  **(β estimate; 95% CI)** | | |
| --- | --- | --- | --- | --- | --- | --- | --- | --- | --- |
|  | **β** | **LL** | **UL** | **β** | **LL** | **UL** | **β** | **LL** | **UL** |
| **AUDIT Total** | 0.00 | -0.03 | 0.03 | 0.00 | -0.03 | 0.03 | 0.03* | 0.00 | 0.06 |
| **APOE4** | 0.06 | -0.17 | 0.30 | 0.06 | -0.17 | 0.30 | 0.10 | -0.07 | 0.27 |
| **Age** | 0.00 | -0.01 | 0.01 | 0.00 | -0.01 | 0.01 | 0.00 | -0.01 | 0.00 |
| **Sex (ref: Male)** | 0.04 | -0.11 | 0.18 | 0.04 | -0.11 | 0.18 | 0.14* | 0.03 | 0.26 |
| **Income** | 0.02 | -0.03 | 0.07 | 0.02 | -0.03 | 0.07 | 0.02 | -0.02 | 0.06 |
| **Education** | 0.00 | -0.01 | 0.02 | 0.00 | -0.01 | 0.02 | 0.02* | 0.00 | 0.05 |
| **GDS Score** | -0.01* | -0.02 | 0.00 | -0.01* | -0.02 | 0.00 | -0.01* | -0.02 | 0.00 |
| **Physical Activity**  **(RAPA 1 Total)** | 0.03 | -0.02 | 0.07 | 0.03 | -0.02 | 0.07 | 0.02 | -0.02 | 0.06 |
| **Physical Activity**  **(RAPA 2 Total)** | 0.02 | -0.03 | 0.08 | 0.02 | -0.03 | 0.08 | 0.05 | 0.00 | 0.09 |
| **BMI** | 0.00 | -0.01 | 0.01 | 0.00 | -0.01 | 0.01 | 0.00 | -0.01 | 0.01 |
| **Diabetes** | 0.02 | -0.12 | 0.17 | 0.02 | -0.12 | 0.17 | -0.12 | -0.30 | 0.06 |
| **Hypertension** | -0.10 | -0.25 | 0.04 | -0.10 | -0.25 | 0.04 | -0.13 | -0.24 | -0.01 |
| **Dyslipidemia** | -0.06 | -0.21 | 0.09 | -0.06 | -0.21 | 0.09 | 0.04* | -0.08 | 0.16 |
| **CVD** | 0.12 | -0.17 | 0.41 | 0.12 | -0.17 | 0.41 | -0.12 | -0.30 | 0.07 |
| **PC1** | 0.03 | -0.66 | 0.73 | 0.03 | -0.66 | 0.73 | 0.41 | -1.04 | 1.85 |
| **PC2** | 0.29 | -0.42 | 0.99 | 0.29 | -0.42 | 0.99 | -0.31 | -0.73 | 0.11 |
| **PC3** | 0.14 | -1.23 | 1.51 | 0.14 | -1.23 | 1.51 | -0.19 | -0.86 | 0.48 |
| **PC4** | -0.04 | -0.79 | 0.72 | -0.04 | -0.79 | 0.72 | -0.26 | -0.85 | 0.33 |
| **PC5** | 0.10 | -0.14 | 0.34 | 0.10 | -0.14 | 0.34 | 0.07 | -0.06 | 0.20 |
| **AUDIT:APOE4** | 0.04 | -0.03 | 0.10 | 0.04 | -0.03 | 0.10 | -0.05* | -0.09 | -0.01 |
| ***FDR p_value^a^*** | 0.46 |  |  | 0.80 |  |  | 0.07 |  |  |

* p-value < 0.05 | ** p-value < 0.01 | *** p-value < 0.001

FDR p_value^a^: p-value for the interaction term after FDR adjustment
