# Supplementary material for: Alcohol use and APOE ε4 interaction with cognitive domains among American adults from diverse racial/ethnic groups: A HABS-HD study
Source: Alzheimers Dement Behav Socioecon Aging. Author manuscript; Available in PMC 2026 Jun 1. (PMC13220920; doi:10.1002/bsa3.70077)
Supplement: S1 [file NIHMS2169993-supplement-S1.docx]

**Supplementary Tables 2**

**Supplementary Table 2a. Results of Sensitivity Analysis 2 for categorized AUDIT scores as primary predictor: Overall analysis across cognitive domains**

|  | **Episodic Memory**  **(β estimate; 95% CI)** | | | **Executive Function**  **(β estimate; 95% CI)** | | | **Processing Speed**  **(β estimate; 95% CI)** | | | **Language**  **(β estimate; 95% CI)** | | |
| --- | --- | --- | --- | --- | --- | --- | --- | --- | --- | --- | --- | --- |
|  | **β** | **LL** | **UL** | **β** | **LL** | **UL** | **β** | **LL** | **UL** | **β** | **LL** | **UL** |
| **AUDIT_Hazardous**  ***Ref: low-risk*** | -0.05 | -0.25 | 0.14 | -0.13 | -0.35 | 0.09 | -0.02 | -0.22 | 0.18 | 0.01 | -0.19 | 0.21 |
| **APOE4** | -0.05 | -0.12 | 0.03 | 0.03 | -0.05 | 0.11 | -0.04 | -0.11 | 0.04 | 0.02 | -0.06 | 0.09 |
| **Age** | 0.00 | -0.01 | 0.00 | -0.01*** | -0.02 | -0.01 | -0.01*** | -0.02 | -0.01 | 0.00 | -0.01 | 0.00 |
| **Sex (ref: Male)** | 0.49*** | 0.42 | 0.57 | 0.09* | 0.01 | 0.17 | 0.27*** | 0.20 | 0.35 | 0.13*** | 0.05 | 0.20 |
| **Income** | 0.02 | 0.00 | 0.05 | 0.06*** | 0.03 | 0.09 | 0.05*** | 0.03 | 0.08 | 0.02 | 0.00 | 0.05 |
| **Education** | 0.01* | 0.00 | 0.03 | 0.02** | 0.01 | 0.03 | 0.02** | 0.01 | 0.03 | 0.02** | 0.01 | 0.03 |
| **GDS Score** | -0.02*** | -0.03 | -0.01 | -0.02*** | -0.03 | -0.01 | -0.02*** | -0.03 | -0.02 | -0.01*** | -0.02 | -0.01 |
| **PC1** | 0.15 | -0.18 | 0.48 | 0.40* | 0.03 | 0.77 | 0.42* | 0.08 | 0.75 | 0.36* | 0.02 | 0.70 |
| **PC2** | 0.04 | -0.29 | 0.36 | -0.13 | -0.50 | 0.23 | -0.11 | -0.44 | 0.22 | -0.13 | -0.46 | 0.20 |
| **PC3** | -0.11 | -0.68 | 0.45 | 0.17 | -0.47 | 0.80 | 0.08 | -0.50 | 0.66 | -0.19 | -0.76 | 0.39 |
| **PC4** | 0.00 | -0.33 | 0.33 | -0.35 | -0.72 | 0.02 | -0.08 | -0.41 | 0.26 | -0.05 | -0.39 | 0.28 |
| **PC5** | 0.01 | -0.09 | 0.12 | 0.04 | -0.08 | 0.15 | 0.11* | 0.01 | 0.22 | 0.07 | -0.04 | 0.18 |
| **Race/ethnicity**  **(ref: NHB)** |  |  |  |  |  |  |  |  |  |  |  |  |
| **Hispanic** | -0.05 | -0.51 | 0.41 | -0.41 | -0.92 | 0.10 | -0.73* | -1.19 | -0.26 | -0.27 | -0.73 | 0.20 |
| **NHW** | -0.22 | -0.69 | 0.24 | -0.76** | -1.28 | -0.24 | -0.65** | -1.13 | -0.18 | -0.56* | -1.03 | -0.09 |
| **AUDIT:APOE4** | -0.35*** | -0.08 | -0.03 | -0.23 | -0.57 | 0.10 | -0.32* | -0.64 | -0.01 | -0.20 | -0.51 | 0.11 |
| ***FDR p_value^a^*** | 0.25 |  |  | 0.50 |  |  | 0.25 |  |  | 0.50 |  |  |

* p-value < 0.05 | ** p-value < 0.01 | *** p-value < 0.001

FDR p_value^a^: p-value for the interaction term after FDR adjustment

**Supplementary Table 2b. Results of Sensitivity Analysis 2 for categorized AUDIT scores as primary predictor: Stratified analysis for episodic memory**

|  | **NHB (n=549)**  **(β estimate; 95% CI)** | | | **Hispanic (n=679)**  **(β estimate; 95% CI)** | | | **NHW (n=915)**  **(β estimate; 95% CI)** | | |
| --- | --- | --- | --- | --- | --- | --- | --- | --- | --- |
|  | **β** | **LL** | **UL** | **β** | **LL** | **UL** | **β** | **LL** | **UL** |
| **AUDIT_Hazardous**  ***Ref: low-risk*** | -0.04 | -0.53 | 0.45 | -0.17 | -0.44 | 0.11 | 0.08 | -0.27 | 0.44 |
| **APOE4** | -0.01 | -0.14 | 0.12 | 0.02 | -0.13 | 0.17 | -0.10 | -0.21 | 0.01 |
| **Age** | 0.00 | -0.01 | 0.01 | 0.00 | 0.00 | 0.01 | -0.01*** | -0.02 | 0.00 |
| **Sex (ref: Male)** | 0.51*** | 0.35 | 0.66 | 0.42*** | 0.29 | 0.55 | 0.54*** | 0.44 | 0.65 |
| **Income** | -0.01 | -0.06 | 0.04 | 0.02 | -0.02 | 0.07 | 0.04 | 0.00 | 0.08 |
| **Education** | 0.06*** | 0.02 | 0.09 | 0.00 | -0.02 | 0.02 | 0.03* | 0.01 | 0.05 |
| **GDS Score** | -0.02* | -0.03 | 0.00 | -0.02** | -0.03 | -0.01 | -0.03*** | -0.04 | -0.02 |
| **PC1** | 1.41 | -0.71 | 3.52 | 0.10 | -0.56 | 0.77 | -0.75 | -2.20 | 0.70 |
| **PC2** | -1.86 | -4.94 | 1.23 | 0.53 | -0.13 | 1.20 | -0.14 | -0.56 | 0.28 |
| **PC3** | -0.57 | -2.82 | 1.69 | 0.16 | -1.14 | 1.46 | 0.05 | -0.63 | 0.74 |
| **PC4** | -0.54 | -2.85 | 1.76 | -0.06 | -0.78 | 0.66 | -0.06 | -0.66 | 0.53 |
| **PC5** | 0.13 | -0.60 | 0.85 | -0.20 | -0.43 | 0.02 | 0.02 | -0.11 | 0.15 |
| **AUDIT:APOE4** | 0.62* | -1.15 | -0.08 | 0.26 | -0.56 | 1.08 | -0.39 | -0.88 | 0.09 |
| ***FDR p_value^a^*** | 0.15 |  |  | 0.84 |  |  | 0.67 |  |  |

* p-value < 0.05 | ** p-value < 0.01 | *** p-value < 0.001

FDR p_value^a^: p-value for the interaction term after FDR adjustment

**Supplementary Table 2c. Results of Sensitivity Analysis 2 for categorized AUDIT scores as primary predictor: Stratified analysis for executive function**

|  | **NHB (n=549)**  **(β estimate; 95% CI)** | | | **Hispanic (n=679)**  **(β estimate; 95% CI)** | | | **NHW (n=915)**  **(β estimate; 95% CI)** | | |
| --- | --- | --- | --- | --- | --- | --- | --- | --- | --- |
|  | **β** | **LL** | **UL** | **β** | **LL** | **UL** | **β** | **LL** | **UL** |
| **AUDIT_Hazardous**  ***Ref: low-risk*** | -0.33 | -0.86 | 0.20 | -0.17 | -0.50 | 0.16 | 0.00 | -0.39 | 0.40 |
| **APOE4** | -0.02 | -0.15 | 0.12 | 0.11 | -0.06 | 0.29 | 0.01 | -0.11 | 0.12 |
| **Age** | -0.01 | -0.02 | 0.00 | 0.00 | -0.02 | 0.01 | -0.01*** | -0.02 | -0.01 |
| **Sex (ref: Male)** | 0.18* | 0.01 | 0.35 | 0.02 | -0.13 | 0.17 | 0.10 | -0.02 | 0.21 |
| **Income** | 0.05 | 0.00 | 0.10 | 0.10*** | 0.05 | 0.16 | 0.03 | -0.02 | 0.08 |
| **Education** | 0.03 | 0.00 | 0.07 | 0.01 | -0.01 | 0.03 | 0.03* | 0.00 | 0.05 |
| **GDS Score** | -0.02** | -0.04 | -0.01 | -0.02** | -0.03 | -0.01 | -0.03*** | -0.04 | -0.01 |
| **PC1** | -1.36 | -3.64 | 0.92 | 0.01 | -0.78 | 0.79 | -0.02 | -1.62 | 1.57 |
| **PC2** | 2.96 | -0.37 | 6.28 | 0.56 | -0.24 | 1.36 | -0.42 | -0.88 | 0.05 |
| **PC3** | -1.04 | -3.47 | 1.40 | 1.08 | -0.46 | 2.63 | 0.07 | -0.68 | 0.82 |
| **PC4** | 2.02 | -0.47 | 4.50 | -0.62 | -1.47 | 0.24 | -0.63 | -1.28 | 0.02 |
| **PC5** | 0.58 | -0.20 | 1.36 | -0.14 | -0.41 | 0.13 | 0.03 | -0.11 | 0.18 |
| **AUDIT:APOE4** | -0.54 | -1.12 | 0.04 | 0.73 | -0.25 | 1.70 | -0.12 | -0.65 | 0.42 |
| ***FDR p_value^a^*** | 0.27 |  |  | 0.71 |  |  | 0.83 |  |  |

* p-value < 0.05 | ** p-value < 0.01 | *** p-value < 0.001

FDR p_value^a^: p-value for the interaction term after FDR adjustment

**Supplementary Table 2d. Results of Sensitivity Analysis 2 for categorized AUDIT scores as primary predictor: Stratified analysis for processing speed**

|  | **NHB (n=549)**  **(β estimate; 95% CI)** | | | **Hispanic (n=679)**  **(β estimate; 95% CI)** | | | **NHW (n=915)**  **(β estimate; 95% CI)** | | |
| --- | --- | --- | --- | --- | --- | --- | --- | --- | --- |
|  | **β** | **LL** | **UL** | **β** | **LL** | **UL** | **β** | **LL** | **UL** |
| **AUDIT_Hazardous**  ***Ref: low-risk*** | -0.56** | -0.97 | -0.14 | 0.09 | -0.21 | 0.40 | 0.08 | -0.30 | 0.45 |
| **APOE4** | -0.07 | -0.18 | 0.03 | 0.02 | -0.14 | 0.18 | -0.06 | -0.18 | 0.05 |
| **Age** | -0.02*** | -0.03 | -0.01 | -0.01 | -0.02 | 0.00 | -0.01*** | -0.02 | -0.01 |
| **Sex (ref: Male)** | 0.33*** | 0.19 | 0.46 | 0.28*** | 0.14 | 0.42 | 0.24*** | 0.13 | 0.35 |
| **Income** | 0.01 | -0.03 | 0.05 | 0.09*** | 0.04 | 0.14 | 0.04 | -0.01 | 0.08 |
| **Education** | 0.04** | 0.01 | 0.06 | 0.01 | -0.01 | 0.03 | 0.02 | -0.01 | 0.04 |
| **GDS Score** | -0.03*** | -0.04 | -0.02 | -0.02** | -0.03 | 0.00 | -0.03*** | -0.04 | -0.02 |
| **PC1** | -0.66 | -2.45 | 1.12 | -0.07 | -0.81 | 0.66 | -0.60 | -2.13 | 0.92 |
| **PC2** | 1.57 | -1.04 | 4.18 | 0.40 | -0.34 | 1.14 | -0.44 | -0.88 | 0.01 |
| **PC3** | 0.05 | -1.85 | 1.96 | 0.74 | -0.70 | 2.18 | -0.03 | -0.75 | 0.69 |
| **PC4** | 0.67 | -1.28 | 2.62 | -0.23 | -1.03 | 0.57 | -0.24 | -0.86 | 0.38 |
| **PC5** | 0.34 | -0.27 | 0.96 | 0.03 | -0.21 | 0.28 | 0.08 | -0.06 | 0.22 |
| **AUDIT:APOE4** | -0.28 | -0.74 | 0.17 | 0.07 | -0.84 | 0.98 | -0.15 | -0.67 | 0.36 |
| ***FDR p_value^a^*** | 0.45 |  |  | 0.96 |  |  | 0.83 |  |  |

* p-value < 0.05 | ** p-value < 0.01 | *** p-value < 0.001

FDR p_value^a^: p-value for the interaction term after FDR adjustment

**Supplementary Table 2e. Results of Sensitivity Analysis 2 for categorized AUDIT scores as primary predictor: Stratified analysis for language**

|  | **NHB (n=549)**  **(β estimate; 95% CI)** | | | **Hispanic (n=679)**  **(β estimate; 95% CI)** | | | **NHW (n=915)**  **(β estimate; 95% CI)** | | |
| --- | --- | --- | --- | --- | --- | --- | --- | --- | --- |
|  | **β** | **LL** | **UL** | **β** | **LL** | **UL** | **β** | **LL** | **UL** |
| **AUDIT_Hazardous**  ***Ref: low-risk*** | -0.28 | -0.78 | 0.22 | -0.01 | -0.29 | 0.28 | 0.17 | -0.19 | 0.52 |
| **APOE4** | -0.03 | -0.16 | 0.10 | 0.16* | 0.00 | 0.31 | -0.02 | -0.13 | 0.09 |
| **Age** | 0.00 | -0.01 | 0.01 | 0.00 | -0.01 | 0.01 | -0.01 | -0.01 | 0.00 |
| **Sex (ref: Male)** | 0.18* | 0.03 | 0.34 | 0.05 | -0.09 | 0.18 | 0.15** | 0.04 | 0.25 |
| **Income** | 0.00 | -0.05 | 0.05 | 0.02 | -0.03 | 0.07 | 0.03 | -0.01 | 0.07 |
| **Education** | 0.04** | 0.01 | 0.08 | 0.01 | -0.01 | 0.02 | 0.03* | 0.01 | 0.05 |
| **GDS Score** | -0.01 | -0.02 | 0.01 | -0.02** | -0.03 | -0.01 | -0.02** | -0.03 | -0.01 |
| **PC1** | -0.57 | -2.71 | 1.58 | -0.01 | -0.70 | 0.69 | 0.19 | -1.26 | 1.65 |
| **PC2** | 1.14 | -1.99 | 4.26 | 0.30 | -0.40 | 1.00 | -0.34 | -0.76 | 0.08 |
| **PC3** | -0.94 | -3.22 | 1.35 | 0.21 | -1.15 | 1.57 | -0.29 | -0.97 | 0.39 |
| **PC4** | 0.19 | -2.15 | 2.52 | -0.06 | -0.81 | 0.70 | -0.23 | -0.82 | 0.36 |
| **PC5** | -0.32 | -1.06 | 0.41 | 0.12 | -0.11 | 0.36 | 0.05 | -0.08 | 0.19 |
| **AUDIT:APOE4** | -0.21 | -0.76 | 0.33 | 0.40 | -0.45 | 1.26 | -0.26 | -0.75 | 0.22 |
| ***FDR p_value^a^*** | 0.66 |  |  | 0.71 |  |  | 0.83 |  |  |

* p-value < 0.05 | ** p-value < 0.01 | *** p-value < 0.001

FDR p_value^a^: p-value for the interaction term after FDR adjustment
